# The Practical Medicine Series of Ten Books

**Published:** 1903-03

**Authors:** 


THE PRACTICAL MEDICINE SERIES OF TEN BOOKS. comprising ten volumes, on the year's progress in medicine and surgery, issued monthly, under the general editorial charge of Gustavus P. Head, M. D., of the Post-Graduate Medical School, Chicago. General Medicine, edited by Frank Billings, M. S., M. D., Chicago, with the collaboration of S. C. Stanton, M. D. Chicago: The Year-Book Publishers, 40 Dearborn St.

The first and second volumes on general medicine are before the reviewer. The reception of the first in October was most satisfactory. The second in May has given as much satisfaction, which warrants the expectation of large sales of these books.

